# Predictive value of EEG-awakening for behavioral awakening from coma

**DOI:** 10.1186/s13613-015-0094-4

**Published:** 2015-12-21

**Authors:** Xiao-gang Kang, Feng Yang, Wen Li, Chen Ma, Li Li, Wen Jiang

**Affiliations:** Department of Neurology, Xijing Hospital, Fourth Military Medical University, Xi’an, 710032 China

**Keywords:** Coma, Prognosis, Electroencephalogram reactivity, Sleep spindles

## Abstract

**Background:**

A reliable predictor for early recovery of consciousness in comatose patients is of great clinical significance. Here we aimed to investigate the potentially prognostic value of electroencephalogram-reactivity (EEG-R) in combination with sleep spindles, termed EEG-awakening, for behavioral awakening in etiologically diverse comatose patients.

**Methods:**

We performed a prospectively observational study on a sample of patients, all of whom were in coma lasting longer than 3 days. Continuous EEG monitoring was performed for at least 24 h to detect the presence of EEG-R and sleep spindles. We then followed patients for 1 month to determine their subsequent level of consciousness, classifying them as either awakened or non-awakened. Finally, Univariate and multivariate analyses were employed to assess the association of predictors with consciousness recovery.

**Results:**

One hundred and six patients with different etiologies leading to coma were included in the study. Of these, 48 patients (45.3 %) awoke and 58 patients (54.7 %) did not awake in the month after the onset of the study. Of note, 26 patients (24.5 %) had a good neurological outcome, and 31 patients (29.3 %) died. Univariate analysis revealed that the Glasgow Coma Scale (GCS) score, EEG-R, sleep spindles, and EEG-awakening were all associated with one-month awakening. Comparisons of the area under the receiving operator characteristic curve (AUC) showed that EEG-awakening (0.839; 0.757–0.921) was superior to all of the following: EEG-R (0.798; 0.710–0.886), sleep spindles (0.772; 0.680–0.864), and GCS scores (0.720; 0.623–0.818). However, age, gender, etiology, and pupillary light reflex did not correlate significantly with one-month awakening. Further logistic regression analysis showed that only EEG-awakening and GCS scores at study entry were significant independent predictors of awakening and that the prognostic model containing these two variables yielded an outstanding predictive performance with an AUC of 0.903.

**Conclusions:**

EEG-awakening incorporates both EEG-R and sleep spindles and is an excellent predictor for early behavioral awakening in comatose patients. The prognostic model combining EEG-awakening and GCS scores shows an outstanding discriminative power for awakening.

## Background

Coma is a severe disorder of consciousness that is characterized by a deficiency of behavioral responsiveness as well as disturbances in sleep-wake cycles that include extended periods of eye closure and muscle inactivity [1]. It is a leading cause of death and disability, and can result from diffuse bihemispheric cortical or white-matter damage, or from focal brainstem lesions [2]. The most common causes of coma include traumatic brain injury, anoxic encephalopathy, vascular, metabolic, or infectious diseases. Acute coma is a major reason of Neurological Intensive Care Unit (N-ICU) admissions and carries high mortality, and neuro-cognitive morbidity, with patients often suffering from subsequent disability [3, 4]. For therapeutic, ethical, and economic reasons, family members consistently ask when, or if, a patient will wake up, i.e., regain consciousness [5]. Despite improvements in neurocritical care, electrophysiology, and neuroimaging, accurate prediction for consciousness recovery in comatose patients remains challenging [6–8].

Consciousness is a multifaceted concept, but has traditionally been divided into two main components:: arousal (i.e., wakefulness, or vigilance) and awareness (e.g., awareness of the environment and of the self) [2]. Arousal reflects a function of the ascending reticular activating system (ARAS) [9], while awareness is more complicated and has been attributed to the functional integrity of the cerebral cortex and its subcortical connections [10, 11]. Given this stratification, arousal is taken as a prerequisite for awareness [9]. Thus, to achieve a more accurate prediction of consciousness recovery, we need to develop an objective test based on the functional assessment of both ARAS and cortical network integrity.

Electroencephalogram (EEG) is the neurological mainstay of electrophysiological testing and has been regarded as a useful and promising tool in determining the prognosis of comatose patients. Continuous EEG is increasingly used in N-ICU to directly and dynamically monitor brain functioning. Given that EEG reactivity (EEG-R) to external stimuli can be interpreted as a sign of functioning of some cortical areas [12] and that sleep spindles in EEG may reflect the preserved functional integrity of the ARAS [13], we sought to predict awakening in a cohort of etiologically diverse comatose patients by integrating these two parameters.

Corresponding to traditional awakening, which is characterized by the presence of behavioral responsiveness and sleep-wake cycles, we propose a new EEG term: EEG-awakening, as defined by the presence of both EEG-R and sleep spindles. In the following study, we examined the predictive value of EEG-awakening for behavioral awakening in comatose patients.

## Methods

### Patients

We conducted a prospective study that enrolled consecutive comatose patients admitted from March 2009 to March 2013 to the N-ICU at one of the largest hospitals in Northwestern China, Xijing Hospital, Fourth Military Medical University. The ethics committee of Xijing Hospital approved all parameters for the study, including a waiver for informed consent since the study design did not modify usual medical practices. We adhered to a strict definition of “coma” as originally defined by Plum and Posner [14] as: a complete lack of awareness of the environment, no eye opening in response to external stimuli, and no purposeful movement to noxious stimulation. The patients with acute coma for more than 3 days were included in this study. Exclusion criteria were as follows: (1) premorbid history of developmental, psychiatric, or neurological illness resulting in documented functional disabilities up to time of the injury; (2) spinal cord impairment; (3) severe, coexisting systemic disease with a limited life expectancy; (4) EEG showing non-convulsive status epilepticus. All patients received standard intensive care and were followed for 1 month after entry into the study, for which the last-observed-carried-forward principle was designed to deal with missing data at the follow-up as it was needed.

### EEG recording

Bedside video-EEG (Solar 2000 N, Solar Electronic Technologies Co., Ltd, Beijing, China) was performed within 3 days of N-ICU admission. Briefly, an array of 20 scalp electrodes were arranged according to the international 10–20 system and was then continuously recorded for at least 24 h. Sedatives and/or anesthetic agents (dexmedetomidine or midazolam) were discontinued for at least 12 h and no patient was under hypothermic therapy at the time of EEG recordings. EEG findings were categorized according to the presence or absence of the following: (1) EEG-R, defined as a clear, reproducible change in either the background frequency or amplitude following repetitive auditory and nociceptive stimulations. This excluded “stimulus-induced-evolving lateralized rhythmic delta activity” or induction of muscle artifacts alone [15]. (2) Sleep spindles, defined as waxing–waning waveforms with a frequency ranging from 12 to 16 Hz, duration between 0.5 and 2 s, and occurring in the context of EEG activity [1]. (3) EEG-awakening, defined as the presence of both EEG-R and sleep spindles. All EEG recordings were interpreted by two EEG-certified neurologists (J.B. and X.X.) who were blind to all clinical data. Agreement was determined with the unweighted Cohen kappa statistic.

### Predictor variable and outcome definitions

We based our clinical and EEG variables on available observation in all selected patients. As a result, the following variables were systematically assessed for each patient: age, gender, etiology, pupillary light reflex, Glasgow Coma Scale (GCS) score on admission to the N-ICU, EEG-R, and sleep spindles. Pupillary light reflex was observed in response to 1-second stimuli of blue light at 5 cm distance under natural light during daytime in the ward of N-ICU. Patients were evaluated daily for 1 month after entry into the study to determine recovery of consciousness, at which point they either stayed in the N-ICU or were transferred to the neurorehabilitation ward. Recovery of consciousness was defined as the patient’s ability to show a clearly discernible evidence of self or environmental awareness and was assessed according to clinical criteria for minimally conscious state (MCS) and for emergence from MCS [16], confirmed by Coma Recovery Scale–Revised (CRS-R) [17]. In order to improve diagnostic accuracy, each patient was evaluated at least five times by CRS-R. For the purpose of statistical analysis, we classified patients into those who recovered consciousness (awakened group) and those who did not regain consciousness (non-awakened group). Patients who regained consciousness and subsequently died due to other causes were still classified as awakening. Neurological outcomes were also assessed at 1 month after entry into the study using the Glasgow–Pittsburgh cerebral performance categories (GP-CPC) [18]. The performance categories were defined as follows: CPC 1, good cerebral performance; CPC 2, moderate cerebral disability; CPC 3, severe cerebral disability; CPC 4, coma or vegetative state (VS); and CPC 5, death.

### Statistical analyses

Continuous variables were expressed as mean ± standard deviation (normally distributed) or as medians and interquartile ranges (not normally distributed). Categorical variables were expressed as counts and percentages. We compared differences in patient characteristics between those who awoke and those who did not. Depending on the data set, a Student’s *t* test, Fisher Exact test, or Mann–Whitney* U* test was used, respectively. All tests were two-sided and a *p*-value <0.05 was considered statistically significant. Each potential prognosticator of awakening was assessed with C statistics using the area under the receiving operator characteristic (ROC) curve. The outcome predictors for awakening were assessed for sensitivity, specificity, positive predictive value (PPV), negative predictive value (NPV), positive likelihood ratios (+LR), negative likelihood ratios (−LR) and unweighted accuracy. Finally, all variables that were significantly different in the two patient populations were entered into a backward stepwise logistic regression model to identify those that significantly predicted awakening. All statistical analyses were performed using SPSS, version 18.0 (SPSS Inc., Chicago, IL, USA).

## Results

One hundred and six comatose patients were included in the final data set and their characteristics and outcomes are listed in Table 1. The mean (SD) age was 50.9 (20.9) years. Seventy-six patients (71.7 %) were males. The median time between coma onset and EEG recording was 7.5 (IQR 4–17) days. EEG recording lasted a mean of 35 ± 6 h. Coma was caused by one the following: traumatic brain injury (TBI, *n* = 13), anoxia (*n* = 14), stroke (*n* = 51), encephalitis (*n* = 25), and poisoning (*n* = 3). In the 1 month after study onset, 26 patients (24.5 %) had a good neurological outcome (CPC 1–2), 48 patients (45.3 %) awoke, and 31 patients (29.3 %) died.

**Table 1:** Patient characteristics of the 106 patients

Characteristic	Value
Age, year, mean (SD)	50.9 (20.9)
Gender, male, no. (%)	76 (71.7)
GCS score, mean (SD)	5.5 (1.6)
Time from coma onset to EEG recording, days, median (IQR)	7.5 (4–17)
Etiology, no. (%)	
Anoxia	14 (13.2)
Trauma	13 (12.3)
Encephalitis	25 (23.6)
Stroke	51 (48.1)
Poisoning	3 (2.8)
Consciousness at 1 month, no. (%)	
Awakening	48 (45.3)
Non-awakening	58 (54.7)
CPC at 1 month, no. (%)	
Good cerebral performance	19 (17.9)
Moderate cerebral disability	7 (6.6)
Severe neurological disability	22 (20.8)
Coma or vegetative state	27 (25.8)
Dead	31 (29.3)

*GCS* Glasgow coma scale, *SD* standard deviation, *IQR* interquartile range, *CPC* Glasgow–Pittsburgh cerebral performance categories

The clinical and EEG characteristics collected at the start of the study in awakened and non-awakened patients are summarized in Table 2. Agreement between EEG raters (J.B. vs X.X.) for measurements of EEG-R and sleep spindles was good. The kappa coefficient for recognition of EEG-R was 0.89 (95 % CI 0.80–0.98), and sleep spindles were 0.92 (95 % CI 0.85–1.00). When EEG-R was present, 41 out of 56 patients awoke, while only seven out of 50 awoke when it was absent. When sleep spindles were present, 41 out of 59 patients awoke, while only seven out of 47 awoke when they were absent. When EEG-awakening was present, 40 out of 49 patients awoke, while only eight out of 57 awoke when it was absent. Univariate analysis showed that patients were more likely to awaken if they presented with EEG-R, sleep spindles, EEG-awakening, and higher admission GCS scores. In contrast, age (*p* = 0.597), gender (*p* = 0.665), etiology (*p* = 0.149), and pupillary light reflex (*p* = 0.086) did not correlate significantly with one-month awakening.

**Table 2:** Univariate analysis

Characteristic	Awakening (n = 48)	Non-awakening (n = 58)	*p* value
Age	52.06 ± 22.05	49.88 ± 20.27	0.597
Gender			0.665
Male	33	43	
Female	15	15	
Time from coma onset to EEG record, days, (median, IQR)	7 (4–16)	8 (4–20)	0.593
Pupillary light reflex			0.086
Present	45	48	
Absent	3	10	
Etiology			0.149
Anoxia	4	10	
Trauma	8	5	
Encephalitis	8	17	
Stroke	28	23	
Poisoning	1	2	
GCS score, mean ± SD	6.29 ± 1.53	4.79 ± 1.34	<0.0001
EEG-R			<0.0001
Present	41	15	
Absent	7	43	
Sleep spindles			<0.0001
Present	41	18	
Absent	7	40	
EEG-awakening			<0.0001
Yes	40	9	
No	8	49	

*CPC* Glasgow–Pittsburgh cerebral performance categories, *SD* standard deviation, *GCS* Glasgow coma scale, *EEG* electroencephalogram, *EEG-R* electroencephalogram reactivity

**Fig. 1:**
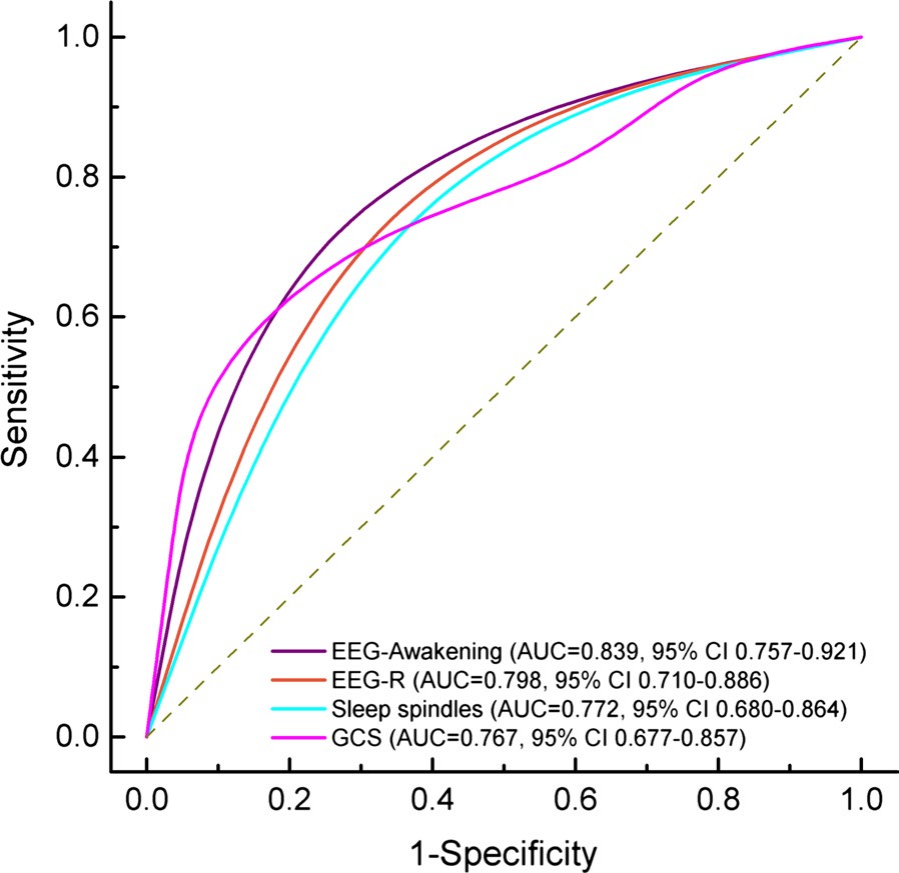
Receiver operator characteristic curves (ROC) showing accuracy for prediction of awakening by GCS, EEG-R, Sleep spindles, and EEG-awakening. The *ordinate axis* shows the test sensitivity, with a range of 0–1.0 (0–100 %). The *abscissa* shows the percentage of false positive results (1-specificity). An area under the ROC curve (AUC) of 1.0 is characteristic of an ideal test, whereas an AUC of 0.5 or less indicates a test with no predictive value

ROC curves were based on the sensitivity and specificity of significant variables in the prediction of awakening and are shown in Fig. 1. The areas under the ROC curves (AUC), which quantitatively estimated the test performance, were analyzed and the results showed that EEG-awakening (0.839; 0.757–0.921) was superior to EEG-R (0.798; 0.710–0.886), sleep spindles (0.772; 0.680–0.864), and GCS score (0.767; 0.677–0.857). As shown in Table 3, while EEG-awakening, EEG-R, and sleep spindles all showed similarly high predictive sensitivity for awakening, only EEG-awakening had both higher specificity (84.5 %) and predictive accuracy (84.0 %), and its +LR (5.4) was also higher than that of EEG-R (3.3) and sleep spindles (2.8).

**Table 3:** Performance of the variables for predicting awakening

	Sensitivity	Specificity	PPV	NPV	+LR	−LR	Predictive accuracy
EEG-R	85.4 (71.6–93.5)	74.1 (60.7–84.4)	73.2 (59.5–83.8)	86.0 (72.6–93.7)	3.3 (2.7–4.0)	0.2 (0.1–0.4)	79.2
Sleep spindles	85.4 (71.6–93.5)	69.0 (55.3–80.1)	69.5 (56.0–80.5)	85.1 (71.1–93.3)	2.8 (2.2–3.4)	0.2 (0.1–0.5)	76.4
EEG-awakening	83.3 (69.2–92.0)	84.5 (72.1–92.2)	81.6 (67.5–90.8)	86.0 (73.7–93.3)	5.4 (4.5–6.4)	0.2 (0.1–0.5)	84.0
GCS	52.1 (37.2–66.7)	93.1 (83.3–98.1)	86.2 (68.3–96.1)	70.1 (58.5–80.1)	7.6 (5.7–10.0)	0.5 (0.2–1.4)	70.8

*EEG* electroencephalogram, *EEG-R* electroencephalogram reactivity, *GCS* Glasgow coma scale, *PPV* positive predictive value, *NPV* negative predictive value, *+LR* positive likelihood ratio, *−LR* negative likelihood ratio

Furthermore, variables that were significantly different between the two groups that were subjected to univariate analysis (GCS, EEG-R, sleep spindles, and EEG-awakening) were entered into a logistic regression model with awakening as the outcome. Only GCS score and the presence of EEG-awakening at the study’s start were significant, independent predictors of awakening (Table 4). The prognostic model containing GCS score (>5) and EEG-awakening yielded an outstanding predictive performance with an AUC of 0.903.

**Table 4:** Multivariable logistic regression model for predicting awakening

Variable	OR (95 % CI)	*p* value	AUC (95 % CI)
GCS (3–5/6–8)	7.607 (2.260–25.609)	0.001	0.903 (0.844–0.962)
EEG-awakening	31.956 (9.510–107.379)	<0.0001	

*OR* odds ratio, *AUC* area under the curve, *GCS* Glasgow coma scale, *EEG* electroencephalogram

## Discussion

In this study, we confirmed the prognostic value of using EEG-R and sleep spindles to determine awakening in a sample of etiologically diverse comatose patients. We found that the combination of EEG-R and sleep spindles, termed EEG-awakening, presented better predictive accuracy for behavioral awakening than if either was used individually. Furthermore, we developed a predictive model containing EEG-awakening and GCS score that showed excellent discriminative power with an AUC of 0.903 for awakening. Thus, our study provides families and clinicians with an important tool to predict early outcomes in comatose patients.

Rather than focusing on how to predict awakening, most previous work has centered on using clinical, neurophysiological, and neuroimaging variables to predict non-awakening [6]. The absence of the somatosensory-evoked potential (SSEP) N20 in comatose patients has traditionally been regarded as a good indicator for the likelihood of non-awakening [6]. However, its presence does not guarantee recovery of consciousness [7, 19].

Event-related potentials (ERPs) can objectively evaluate higher order cortical functions associated with stimulus detection and decision-making and have recently been studied to predict awakening from coma. A meta-analysis has confirmed that the presence of each of the ERP components, such as N100, mismatch negativity (MMN), and P300, is a highly significant predictor for awakening [20]. However, their use in predicting non-awakening has been questioned since (i) the practicality of using ERPs has technical limitations, and (ii) ERP components are not mandatory evoked potentials, even in healthy participants [21]; thus their absence does not predict non-awakening. Moreover, until now, no prognostic model for awakening has yet been proven suitable for generalization across different coma etiologies.

As EEG is a technique that is routinely available in most neurophysiological laboratories, our results support its role as a potential prognostic tool in the assessment of comatose patients with different etiologies. EEG activity reflects the temporal synchronization of cortical pyramidal neurons, which is taken as a neural substrate for human cognition and conscious awareness [22]. After external stimuli, EEG-R represents the neural activity along afferent somatosensory pathways from the ARAS to the cortex. Thus, EEG-R in comatose patients can be interpreted as a sign of the impending recovery of consciousness, in that sensory stimulation produces desynchronized arousal EEG patterns. This would, therefore, suggest that the brain is responsive to the outside world [12].

The prognostic significance of EEG-R in comatose patients was first reported by Fishgold and Mathus [23, 24] in 1959 and later by Synek [25] and Young et al. [26] in comatose patients from various etiologies. Of note, Gutling et al. [27] compared EEG-R with SSEPs and GCS in 50 comatose patients with severe head injury and found that EEG-R alone is an excellent long-term, global outcome predictor, superior to both GCS and SSEPs. Ramachandrannair et al. [28] retrospectively analyzed 33 comatose children with various etiologies, including anoxia, head injury, infection, and stroke, and found that 71.4 % of children with EEG-R had a favorable outcome. Recently, Rossetti et al. [29–31] prospectively studied 111 comatose survivors of cardiac arrest (CA) that had been treated with therapeutic hypothermia and reported that EEG-R was strongly associated with outcome. In this study, we prospectively examined 106 comatose patients with various etiologies, of whom two-thirds were affected by stroke and encephalitis. In order to exclude many of the neurological and non-neurological factors that could affect EEG recording in the hyper-acute stage of coma, we enrolled comatose patients for more than 3 days. Thus, when EEG recordings were performed, their contribution to the overall prognosis was more reliable. As expected, we found that EEG-R was significantly associated with awakening.

The sleep spindle is one of the hallmarks of human stage two sleep and is also one of the few transient EEG events that is unique to sleep [32]. Thus, absence of sleep spindles in coma could imply an absence of sleep elements and the consequent lack of sleep-wake cycles as measured by EEG. Given that human spindle generators are located in the thalamus, it is tempting to hypothesize that the absence of spindles in coma results from the interruption of either the ascending reticular thalamocortical pathway or of the thalamocortical loops [13, 33, 34]. Some earlier studies indicated that sleep spindles carry prognostic information [35]. It was subsequently shown that the presence of spindle after hypoxic or anoxic injury does not always indicate a good outcome and that the absence of spindles has been associated with a poor outcome [36]. A more recent study by Urakami [37] examined spindle activity in the acute, the sub-acute, and the chronic stages of posttraumatic coma, and concluded that spindles may reflect recovery of consciousness in patients following diffuse axonal injuries. Our findings have extended these results from previous studies and confirmed that spindles have a good predictive accuracy for recovery of consciousness in comatose patients with different etiologies.

As already mentioned, EEG-R and sleep spindles involve different anatomical structures for awakening. Thus, it is theoretically possible that combining the two measures would increase predictive power. Support for this possibility is demonstrated by the results of our study. Given that behavioral awakening includes two essential elements, behavioral responsiveness and the sleep-wake cycle, we propose that EEG-awakening is based on the idea that EEG-R and sleep spindles correspond to behavioral response and sleep-wake cycle, respectively. In this study, when EEG-awakening was present, 40 out of 49 patients awoke, while only eight out of 57 awoke when it was absent. These results provide robust evidence that EEG-awakening has excellent predictive accuracy for behavioral awakening. Since the two components of EEG-awakening are easily detectable using scalp EEG, it has the potential to be used as a good prognostic marker for behavioral awakening and may well be an alternative to ERPs in ICUs that lack ERP facilities.

The pupillary light reflex has been used as an important determinant for poor outcome in comatose patients with hypoxic-ischemia [38] or TBI [39, 40] because of its low interobserver variability [41]. However, there have been reports that patients in nontraumatic coma with absent pupillary reflexes still achieved good outcomes [42]. In this study, we noted that the pupillary reflex was not a predictive factor for awakening in comatose patients. However, we do not exclude the possibility that the absence of pupillary reflex is associated with poor outcomes.

The GCS has been widely adopted as a simple method to quantitatively express the clinically observed features of consciousness [43]. Many studies have shown that a patient’s GCS score may provide information in identifying those with either a favorable or unfavorable neurological outcome after cardiac arrest or TBI [39, 44, 45]. Recently, Goodman et al. retrospectively studied 51 comatose patients with intracerebral hemorrhage and found that their GCS score was the predominant initial predictor for early awakening [46]. Fischer et al. [47] prospectively studied 346 comatose patients with various etiologies, including stroke, trauma, anoxia, encephalitis and complications of neurosurgery, and found that GCS on admission was correlated with awakening in comatose patients with different etiologies. Moreover, they also found that etiology was a prognostic factor for awakening but with differing modalities for each etiology. In this study, we collected clinical data from 106 comatose patients, most of who had etiologies of stroke and encephalitis, and demonstrated that patients with higher admission GCS scores were more likely to awaken. The highest +LR (7.6) was predicting awakening with GCS; however, its −LR was also the highest. Comparisons of the ROC-AUC showed that GCS scores (0.720; 0.623–0.818) were inferior to all of the following: EEG-awakening (0.839; 0.757–0.921), EEG-R (0.798; 0.710–0.886), and sleep spindles (0.772; 0.680–0.864). Accordingly, the predictive accuracy of GCS (70.8 %) was lower than that of EEG-awakening (84.0 %), EEG-R (79.2 %), and sleep spindles (76.4 %). Furthermore, using multivariate logistic regression analysis, we established a prognostic model incorporating EEG-awakening with GCS scores and found that this model had outstanding performance in predicting awakening from coma. The ROC-AUC of the model (0.903; 0.844–0.962) was superior to EEG-awakening and GCS scores, respectively. This suggests that a multimodal prognostication approach is best when determining coma patient outcomes.

Previous studies showed that the prognosis in comatose patients with various etiologies is different. Post-anoxic coma is apt to have a poor outcome. Of comatose patients after cardiac arrest, 40–66 % never regained consciousness [48]. In our study, they represented only 14 of the 106 patients included and four (28.6 %) patients awoke. Traumatic coma would have better outcome than post-anoxic patients, and a majority of these patients ultimately recover consciousness and up to 20 % of traumatic coma eventually achieve household independence [49]. Although the prognosis in various etiologic coma is different, the outcome of patients with the same etiology may be very different owing to the difference in severity of brain injury. Recent studies suggested that brain structural changes [50] and residual brain function [48] were associated with the outcome in comatose patients. By using cEEG to detect their residual brain function, we hope to explore a more widely applicable predictor for comatose patients.

However, the present study raises further questions and has some limitations. First, the results obtained from our limited patient group need to be confirmed in a larger, multicenter analysis. Moreover, we do not provide data on long-term outcomes for the patients in our study population. Second, the time from coma onset to EEG recording was not uniform in this study; thus its IQR was very wide. It is very hard to perform the video EEG for all the comatose patients in a consistent time. In some patients, especially traumatic or postoperative ones, electrodes could not be placed on the scalp in the early time after admission; thus the time between the video EEG recording and coma onset was relatively long in these patients. There was no selection of cases based on the time elapsed since coma onset because we wanted the study to be as temporally close to a clinical setting as possible. Third, although the half-life of midazolam is very short (1.5–2.5 h), it may cumulate with a markedly longer half-life in case of continuous infusion. Twelve hours off midazolam after a prolonged infusion may be not enough to completely clear the drug in some patients, which may have an impact on the EEG monitoring to some extent. Fourth, the heterogeneity of etiologies constituted the cohort of patients in our study. Previous studies showed that the prognosis in comatose patients with various etiologies is different. Post-anoxic coma is apt to have a poor outcome, and traumatic coma would have better outcome than post-anoxic patients. It would be optimal to carry out an exploratory analysis on single etiology. Given the relatively small sample size of this study, we did not further explore the performance of our model among the different etiologies separately. Future large sample study is needed. Finally, visual EEG analysis is time-consuming, operator-dependent, non-quantitative, and lacks standardization. In future studies, it will be worthwhile to use automated analysis techniques to extract only the most important quantitative EEG variables.

## Conclusions

We propose a new term of EEG-awakening and demonstrate that it is an excellent predictor for behavioral awakening from coma. Furthermore, we develop a predictive model combining EEG-awakening and GCS scores that has an outstanding prognostic value. However, it should be stated that our technique does not yet have the accuracy for meaningful decision-making at the individual patient level. This is because prognostic estimates always represent probabilities and not absolute certainties on the actual outcome at any given time. Thus, no judgment could be made on the basis of our technique as to whether the patients continue to receive intensive treatment or not.

## Abbreviations

EEG-R: electroencephalogram reactivity
GCS: Glasgow coma scale
MCS: minimally conscious state
CRS-R: Coma Recovery Scale–Revised
CPC: Glasgow–Pittsburgh cerebral performance categories
SSEPs: somatosensory evoked potentials
ERPs: event-related potentials
ARAS: ascending reticular activating system
ROC: receiver operating characteristic curve
AUC: An area under the ROC curve
PPV: positive predictive value
NPV: negative predictive value

## References

[1] Landsness E, Bruno MA, Noirhomme Q, Riedner B, Gosseries O, Schnakers C (2011). Electrophysiological correlates of behavioural changes in vigilance in vegetative state and minimally conscious state. Brain.

[2] Laureys S, Owen AM, Schiff ND (2004). Brain function in coma, vegetative state, and related disorders. Lancet Neurol.

[3] Trubel HK, Novotny E, Lister G (2003). Outcome of coma in children. Curr Opin Pediatr.

[4] Horsting MW, Franken MD, Meulenbelt J, van Klei WA, de Lange DW (2015). The etiology and outcome of non-traumatic coma in critical care: a systematic review. BMC Anesthesiol.

[5] Peigne V, Chaize M, Falissard B, Kentish-Barnes N, Rusinova K, Megarbane B (2011). Important questions asked by family members of intensive care unit patients. Crit Care Med.

[6] Wijdicks EF, Hijdra A, Young GB, Bassetti CL, Wiebe S (2006). Practice parameter: prediction of outcome in comatose survivors after cardiopulmonary resuscitation (an evidence-based review): report of the Quality Standards Subcommittee of the American Academy of Neurology. Neurology.

[7] Houlden DA, Taylor AB, Feinstein A, Midha R, Bethune AJ, Stewart CP, Schwartz ML (2010). Early somatosensory evoked potential grades in comatose traumatic brain injury patients predict cognitive and functional outcome. Crit Care Med.

[8] Puttgen HA, Geocadin R (2007). Predicting neurological outcome following cardiac arrest. J Neurol Sci.

[9] Young GB (2009). Coma. Ann N Y Acad Sci.

[10] Bernat JL (2006). Chronic disorders of consciousness. Lancet.

[11] Laureys S (2005). The neural correlate of (un)awareness: lessons from the vegetative state. Trends Cogn Sci.

[12] Koenig MA, Kaplan PW (2013). Clinical neurophysiology in acute coma and disorders of consciousness. Semin Neurol.

[13] Cologan V, Schabus M, Ledoux D, Moonen G, Maquet P, Laureys S (2010). Sleep in disorders of consciousness. Sleep Med Rev.

[14] Plum F, Posner JB (1972). The diagnosis of stupor and coma. Contemp Neurol Ser.

[15] Rossetti AO, Carrera E, Oddo M (2012). Early EEG correlates of neuronal injury after brain anoxia. Neurology.

[16] Giacino JT, Ashwal S, Childs N, Cranford R, Jennett B, Katz DI (2002). The minimally conscious state: definition and diagnostic criteria. Neurology.

[17] Giacino JT, Kalmar K, Whyte J (2004). The JFK Coma Recovery Scale-Revised: measurement characteristics and diagnostic utility. Arch Phys Med Rehabil.

[18] Longstreth WT, Diehr P, Inui TS (1983). Prediction of awakening after out-of-hospital cardiac arrest. N Engl J Med.

[19] Robinson LR, Micklesen PJ, Tirschwell DL, Lew HL (2003). Predictive value of somatosensory evoked potentials for awakening from coma. Crit Care Med.

[20] Daltrozzo J, Wioland N, Mutschler V, Kotchoubey B (2007). Predicting coma and other low responsive patients outcome using event-related brain potentials: a meta-analysis. Clin Neurophysiol.

[21] Guerit JM (2004). Prognostic contribution for potentials evoked in unit of intensive care. Ann Fr Anesth Reanim.

[22] Klimesch W (1999). EEG alpha and theta oscillations reflect cognitive and memory performance: a review and analysis. Brain Res Brain Res Rev.

[23] Fishgold H, Mathis P (1959). Obnubilations comas et stupeurs: études électroencephalographiques. Electroencephalogr Clin Neurophysiol.

[24] Thenayan EA, Savard M, Sharpe MD, Norton L, Young B (2010). Electroencephalogram for prognosis after cardiac arrest. J Crit Care.

[25] Synek VM (1990). Revised EEG coma scale in diffuse acute head injuries in adults. Clin Exp Neurol.

[26] Young GB, Kreeft JH, McLachlan RS, Demelo J (1999). EEG and clinical associations with mortality in comatose patients in a general intensive care unit. J Clin Neurophysiol.

[27] Gutling E, Gonser A, Imhof HG, Landis T (1995). EEG reactivity in the prognosis of severe head injury. Neurology.

[28] Ramachandrannair R, Sharma R, Weiss SK, Cortez MA (2005). Reactive EEG patterns in pediatric coma. Pediatr Neurol.

[29] Rossetti AO, Urbano LA, Delodder F, Kaplan PW, Oddo M (2010). Prognostic value of continuous EEG monitoring during therapeutic hypothermia after cardiac arrest. Crit Care.

[30] Juan E, Novy J, Suys T, Oddo M, Rossetti AO (2015). Clinical evolution after a non-reactive hypothermic EEG following cardiac arrest. Neurocrit Care.

[31] Tsetsou S, Oddo M, Rossetti AO (2013). Clinical outcome after a reactive hypothermic EEG following cardiac arrest. Neurocrit Care.

[32] De Gennaro L, Ferrara M (2003). Sleep spindles: an overview. Sleep Med Rev.

[33] Bartho P, Slezia A, Matyas F, Faradzs-Zade L, Ulbert I, Harris KD, Acsady L (2014). Ongoing network state controls the length of sleep spindles via inhibitory activity. Neuron.

[34] Brown RE, McKenna JT (2015). Turning a Negative into a Positive: ascending GABAergic Control of Cortical Activation and Arousal. Front Neurol.

[35] Rumpl E, Prugger M, Bauer G, Gerstenbrand F, Hackl JM, Pallua A (1983). Incidence and prognostic value of spindles in post-traumatic coma. Electroencephalogr Clin Neurophysiol.

[36] Hulihan JF, Syna DR (1994). Electroencephalographic sleep patterns in post-anoxic stupor and coma. Neurology.

[37] Urakami Y (2012). Relationship between, sleep spindles and clinical recovery in patients with traumatic brain injury: a simultaneous EEG and MEG study. Clin EEG Neurosci.

[38] Kamps MJ, Horn J, Oddo M, Fugate JE, Storm C, Cronberg T (2013). Prognostication of neurologic outcome in cardiac arrest patients after mild therapeutic hypothermia: a meta-analysis of the current literature. Intensive Care Med.

[39] Perel P, Arango M, Clayton T, Edwards P, Komolafe E, Poccock S (2008). Predicting outcome after traumatic brain injury: practical prognostic models based on large cohort of international patients. BMJ.

[40] Steyerberg EW, Mushkudiani N, Perel P, Butcher I, McHugh GH (2008). Predicting outcome after traumatic brain injury: development and international validation of prognostic scores based on admission characteristics. PLoS Med.

[41] van den Berge JH, Schouten HJ, Boomstra S, Drunen Littel S, Braakman R (1979). Interobserver agreement in assessment of ocular signs in coma. J Neurol Neurosurg Psychiatry.

[42] Brendler SJ, Selverstone B (1970). Recovery from decerebration. Brain.

[43] Teasdale G, Jennett B (1974). Assessment of coma and impaired consciousness. A practical scale. Lancet.

[44] Schefold JC, Storm C, Kruger A, Ploner CJ, Hasper D (2009). The Glasgow Coma Score is a predictor of good outcome in cardiac arrest patients treated with therapeutic hypothermia. Resuscitation.

[45] Sandroni C, Cariou A, Cavallaro F, Cronberg T, Friberg H, Hoedemaekers C (2014). Prognostication in comatose survivors of cardiac arrest: an advisory statement from the European Resuscitation Council and the European Society of Intensive Care Medicine. Resuscitation.

[46] Goodman D, Kasner SE, Park S (2013). Predicting early awakening from coma after intracerebral hemorrhage. Front Neurol.

[47] Fischer C, Luaute J, Adeleine P, Morlet D (2004). Predictive value of sensory and cognitive evoked potentials for awakening from coma. Neurology.

[48] Hofmeijer J, Beernink TM, Bosch FH, Beishuizen A, Tjepkema-Cloostermans MC, van Putten MJ (2015). Early EEG contributes to multimodal outcome prediction of postanoxic coma. Neurology.

[49] Edlow BL, Giacino JT, Wu O (2013). Functional MRI and outcome in traumatic coma. Curr Neurol Neurosci Rep.

[50] Kowalski RG, Buitrago MM, Duckworth J, Chonka ZD, Puttgen HA, Stevens RD, Geocadin RG (2015). Neuroanatomical predictors of awakening in acutely comatose patients. Ann Neurol.

